# Liquid‒liquid phase separation: roles and implications in future cancer treatment

**DOI:** 10.7150/ijbs.81521

**Published:** 2023-08-06

**Authors:** Zheran Liu, Zijian Qin, Yingtong Liu, Xi Xia, Ling He, Na Chen, Xiaolin Hu, Xingchen Peng

**Affiliations:** 1 Department of Biotherapy and National Clinical Research Center for Geriatrics, Cancer Center, West China Hospital, Sichuan University, Chengdu 610041, Sichuan, China.; 2 Chengdu University of Traditional Chinese Medicine, Chengdu 610041, Sichuan, China.; 3 Shanghai ETERN Biopharma Co., Ltd., Shanghai, China.; 4 School of Pharmacy, Chengdu Medical College, Xindu Avenue No 783, Chengdu, 610500, Sichuan Province, China.; 5 West China School of Nursing, West China Hospital, Sichuan University, Chengdu 610041, Sichuan, China.

**Keywords:** liquid‒liquid phase separation, biomolecular condensates, hematologic neoplasms, solid tumors

## Abstract

Liquid‒liquid phase separation (LLPS) is a phenomenon driven by weak interactions between biomolecules, such as proteins and nucleic acids, that leads to the formation of distinct liquid-like condensates. Through LLPS, membraneless condensates are formed, selectively concentrating specific proteins while excluding other molecules to maintain normal cellular functions. Emerging evidence shows that cancer-related mutations cause aberrant condensate assembly, resulting in disrupted signal transduction, impaired DNA repair, and abnormal chromatin organization and eventually contributing to tumorigenesis. The objective of this review is to summarize recent advancements in understanding the potential implications of LLPS in the contexts of cancer progression and therapeutic interventions. By interfering with LLPS, it may be possible to restore normal cellular processes and inhibit tumor progression. The underlying mechanisms and potential drug targets associated with LLPS in cancer are discussed, shedding light on promising opportunities for novel therapeutic interventions.

## 1. Introduction

Cancer development is an intricate process driven by randomly occurring mutations and genetic alterations. The complexities of cancer biology involve various factors, such as dysregulated signaling pathways leading to excessive cell proliferation, mechanisms that confer resistance to cell death, evasion of growth suppression mechanisms, sustained replicative potential, promotion of angiogenesis, and cellular invasive properties responsible for tissue invasion and metastasis 1,2. Despite the substantial progress in identifying the primary drivers of genetic mutations in cancer development, the precise pathological mechanisms underlying these processes are incompletely elucidated 3.

**Figure 1:**
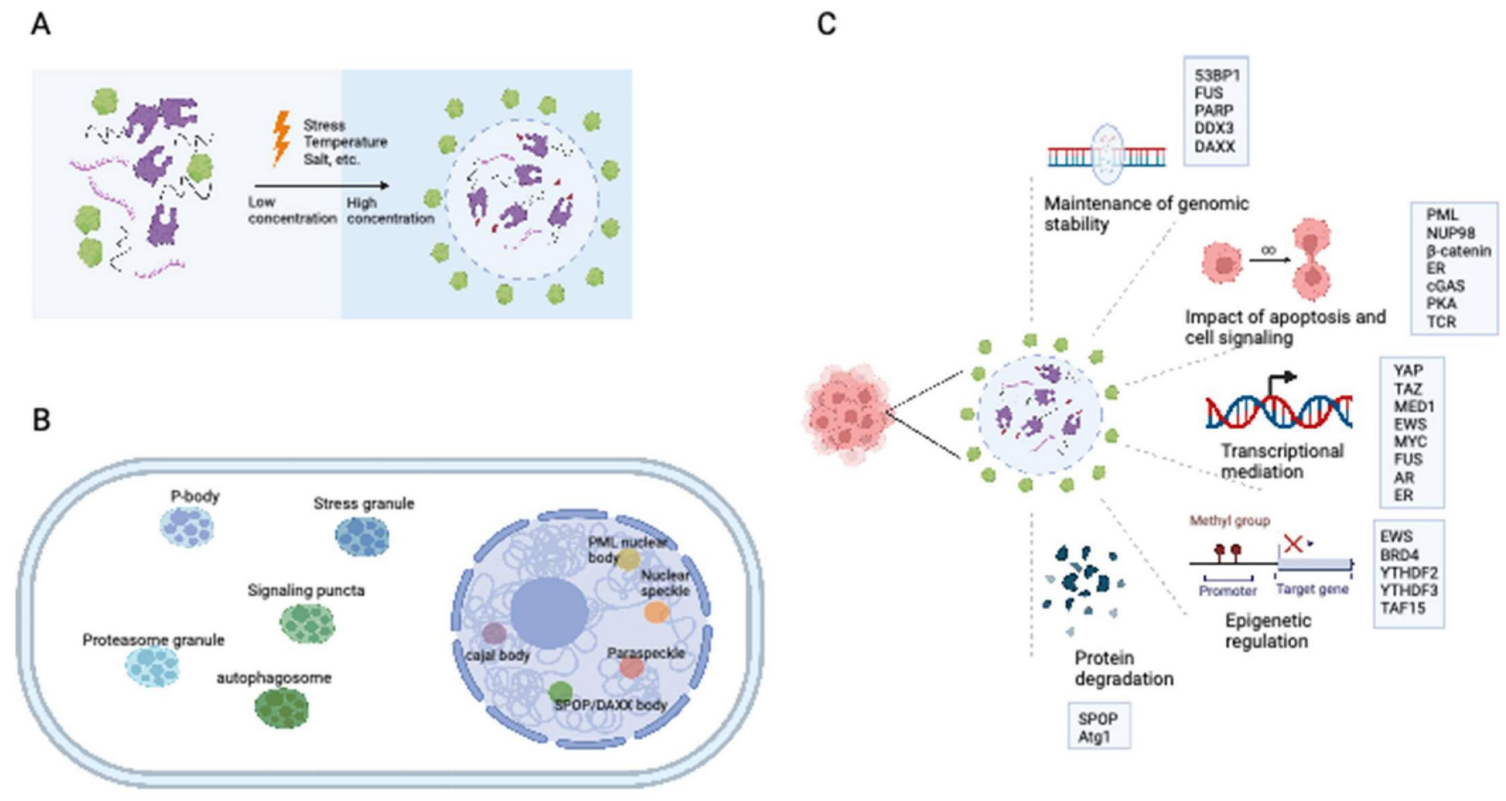
** The formation of biomolecular condensates through LLPS.** (A) The process of LLPS. Weakly multivalent interaction exists among scaffold proteins or between proteins and nucleic acids. External or internal stress can lead to LLPS. Biomolecules and their intercalating substrates maintain high concentrations in condensates while other proteins or substrates are excluded. (B) Nuclear and cytoplasmic condensates in eukaryotic cells. (C) Cellular functions and proteins that related to LLPS in cancer cells. IDR: intrinsically disordered regions; LLPS: liquid-liquid phase separation. AR: androgen receptor; ER: Estrogen receptor.

Eukaryotic cells are equipped with organelles that generate distinct chemical microenvironments to facilitate various biological processes. Through spatial segregation, various proteins and macromolecules are compartmentalized within subcellular structures, allowing the precise spatial and temporal control of complex biochemical reactions 4. In addition to membrane-bound organelles, membraneless structures, such as stress granules (SGs), Cajal bodies, and promyelocytic leukemia (PML) nuclear bodies (NBs),are essential for maintaining the normal functionality of eukaryotic cells. Liquid‒liquid phase separation (LLPS) is a cellular phenomenon characterized by the spontaneous segregation of macromolecules into dense and dilute phases, resulting in the formation of biomolecular condensates (Figure 1A) 5,6. These condensates generate a heterogeneous cellular environment, selectively enriching proteins and nucleic acids. They possess unique properties that facilitate biomolecule concentration and organization (Figure 1B) 7,8.

The establishment of interaction networks involving multivalent proteins or nucleic acids is crucial for LLPS and is facilitated primarily by peptides with folded modular domains, intrinsically disordered regions (IDRs), or polymerizing domains 9. Cancer-associated proteins and mutations can modulate the abundance and formation of condensates by impacting LLPS, thereby driving aberrant cellular activities and promoting tumorigenesis (Figure 1C) 10-13. This review presents an overview of the impact of LLPS on the development and progression of diverse cancer types, including hematological malignancies and solid tumors (Table 1). Furthermore, we discuss potential therapeutic approaches targeting LLPS that could be employed in cancer treatment strategies.

## 2. LLPS in leukemia and myeloma

### 2.1 NUcleoPorin 98 (NUP98) fusion protein and acute myeloid leukemia (AML)

LLPS is a pivotal molecular alteration that promotes tumorigenicity. Recurrent gene fusions involving IDRs and chromatin-binding proteins are commonly detected in various cancer types, notably, leukemia and sarcoma 14-16.

NUP98 is a constituent of the nuclear pore complex that facilitates macromolecule transport between the nucleus and cytoplasm 17. The discovery of the NUP98-HOXA9 fusion in AML patients with the t^7^;^11^(p15;p15) translocation marked the first identification of NUP98 rearrangement. Since that discovery, more than 31 distinct fusion partner genes of NUP98 have been identified in various hematological malignancies, including T-cell acute lymphoblastic leukemia 18, chronic myeloid leukemia (CML) 19, and AML 20.

**Figure 2:**
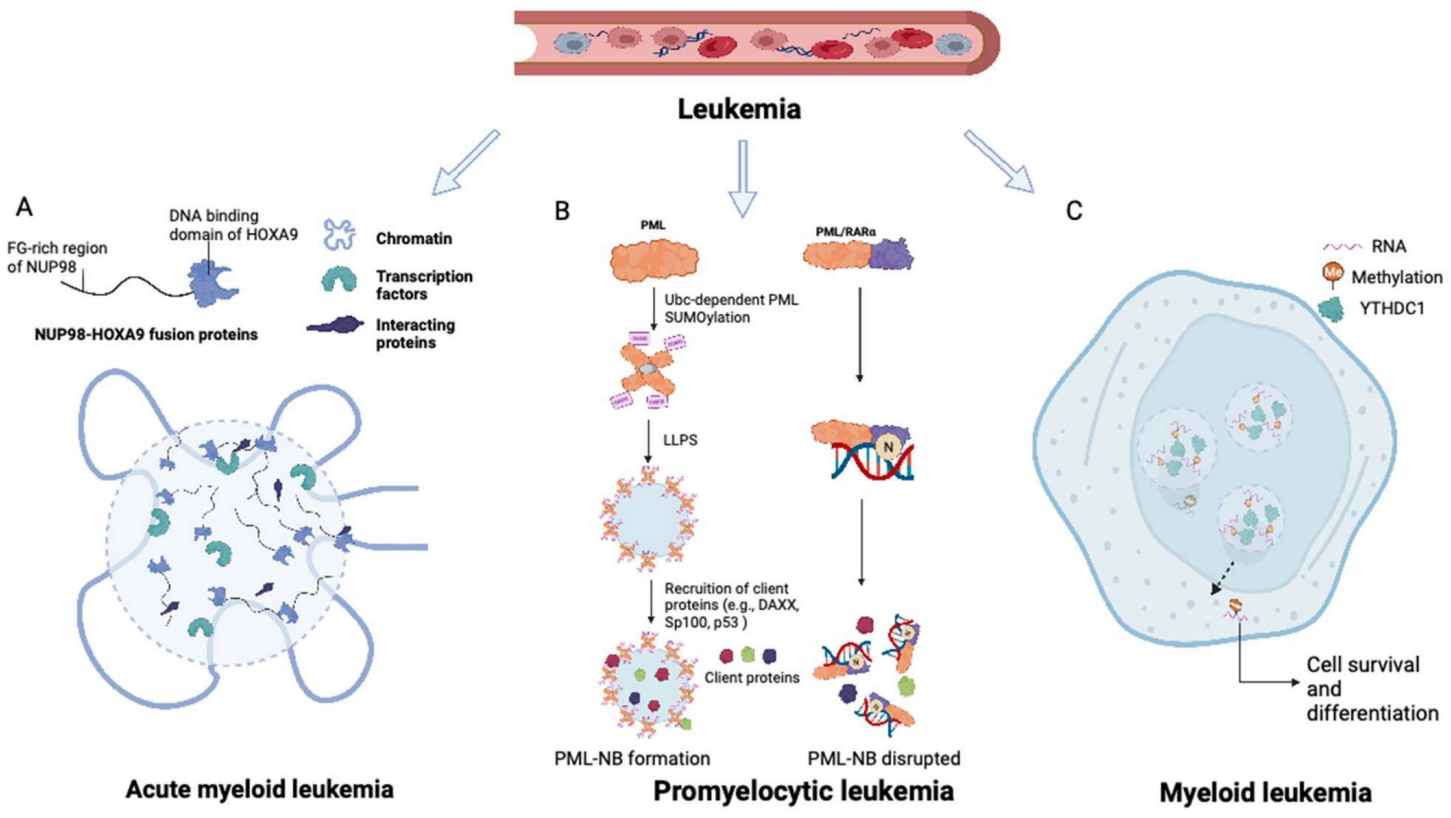
** Roles of LLPS in leukemia and myeloma.** (A) NUP98-HOXA9 fusion protein forms transcription condensates and leads to the activation of leukemogenesis-related genes. (B) The formation of PML NB was interrupted in promyelocytic leukemia with PML-RARα fusions. The disruption of PML NB in APL is mainly caused by the neddylation of the RARα part at the PML-RARα fusions, promoting its DNA-binding competency and hampering the further LLPS process. (C) M6A-mRNA needs YTHDC1 to undergo LLPS and form YTHDC1-m6A condensates in the nucleus to keep cell survival and maintain the undifferentiated states. YTHDC1-m6A condensates accumulate in acute myeloid leukemia, while if YTHDC1 is depleted in the AML cells, cell death and differentiation are promoted. LLPS: liquid-liquid phase separation; PML: promyelocytic leukemia. NB: nuclear body.

The intrinsically disordered FG domain within the NUP98 IDR-containing N-terminus has been shown to undergo spontaneous phase separation, forming FG particles (Figure 2A). These dense molecules repel inert macromolecules while allowing the entry of nuclear transport receptors carrying necessary cargo 21. NUP98 fusion proteins induce substantial modifications in the composition of biomolecular condensates, exhibiting characteristics distinct from those of native NUP98. Through associations with various cofactors, NUP98 fusion proteins modulate transcriptional changes. The FG domain of the NUP98 fusion protein is important for its specific subcellular localization and the induction of downstream leukemia-associated gene expression through LLPS 22. This process is essential for establishing a temporospatial subcellular environment, promoting chromatin remodeling, and altering gene expression patterns. Nuclear puncta containing multiple NUP98 fusion proteins (e.g., NUP98-NSD1, NUP98-HOXA9, and NUP98-HD) have been observed 23-25. It can thus be inferred that NUP98 fusion proteins associate with transcriptional cofactors in biomolecular condensates, which function as transcription centers to promote aberrant transcriptional programs 26.

Ahn et al. conducted a study demonstrating the essential LLPS-related role of IDRs in NUP98-HOXA9 fusion proteins 27. These authors elucidated the role of NUP98-HOXA9 phase separation in enhancing the ability of chimeric transcription factors to interact with and bind to specific chromatin domains, thus enabling long-range interactions between oncogene promoters and enhancer elements 27. Chandra et al. further showed that LLPS could drive the formation of NUP98-HOX9 nuclear puncta. The intermolecular interactions in the FG-repeat domain of NUP98-HOX9 and the heterotypic DNA binding regulated by the homeodomain of HOX9 are the driving forces of LLPS *in vitro* 28. The LLPS dynamics of NUP98-HOXA9 exhibited a direct correlation with the upregulation of genes associated with cell transformation and leukemogenesis 29. Similarly, fusion proteins such as NUP98-PRRX1, NUP98-KDM5A, and NUP98-LNP1 were observed to form nuclear condensates and induce the transformation of hematopoietic stem cells 28.

In summary, NUP98 fusion proteins play a leukemogenic role in hematopoietic cells, partially through LLPS. NUP98 fusion proteins are recognized as significant risk factors for leukemia. Thus, targeting NUP98 fusion proteins and NUP98-mediated LLPS constitutes a promising therapeutic approach for AML. Heikamp et al. demonstrated that inhibiting the menin-MLL1 protein complex using VTP50469 could inhibit leukemogenesis in NUP98-rearranged leukemia models by disrupting interactions between NUP98 fusion proteins and chromatin. Additionally, administration of VTP50469 resulted in improved survival outcomes in mice with NUP98-rearranged leukemias 29, highlighting the potential of VTP50469 as a targeted agent for the treatment of patients with NUP98-rearranged leukemias.

### 2.2 PML NBs and PML

PML NBs are phase-separated, stress-sensitive nuclear condensates implicated in multiple cellular functions, such as transcriptional regulation, cell proliferation control, stem cell self-renewal maintenance, and protein modification 30,31. The PML protein is an important component of and scaffold for PML NBs 32. It contains a conserved N-terminal RING finger/B-box/coiled-coil (RBCC) domain that governs the assembly of PML NBs 33-35, while its C-terminus contains a SUMO-interacting motif (SIM) for binding sumoylated proteins 36. These modular domains and interaction motifs are essential for driving LLPS and the formation of PML NBs 37. Extensive evidence indicates the tumor-suppressive functions of PML NBs mediated through multiple mechanisms, including sequestering proteins, serving as hubs for posttranslational modifications (PTMs), and regulating protein interactions 38,39. Furthermore, PML NBs influence critical cellular pathways such as the P53 and AKT signaling and DNA damage repair pathways 38,39.

In acute promyelocytic leukemia (APL), the PML gene undergoes a chromosomal translocation with retinoic acid receptor-α (RARα), resulting in the formation of the PML-RARα fusion protein, which is centrally involved in the initiation and progression of APL 40. PML-RARα fusions are found in over 95% of APL patients 40. Fusion of the PML gene with RARα in APL has been associated with two primary breakpoints, specifically, between exons 3-4 and exons 6-7, as evidenced by previous studies 41-43. Aberrant LLPS of PML-RARα resulting from neddylation is responsible for the impairment of PML NB assembly in APL. The neddylation-mediated enhancement of DNA binding of PML-RARα to the RARα domain interferes with LLPS of the PML domain, thereby hindering the assembly of PML NBs (Figure 2B) 44.

Potential therapeutic strategies to restore PML NBs have been explored. Retinoic acid (RA) and arsenic trioxide therapies have shown promise in inducing the re-formation of PML NBs and restoration of LLPS; these therapies promote apoptosis through activation of p53, localization of HIRA, and degradation of unwanted proteins 45. Various synthetic molecules such as emodin, MLN4924, and TSA, as well as natural compounds such as H_2_O_2_, EGF, and SFN, have also been found to increase the PML protein abundance 46-48. Notably, the neddylation inhibitor MLN4924 has demonstrated efficacy in suppressing the development of APL cells.

Current research efforts are focused on identifying the most effective combination regimens that maximize anticancer activity by enhancing PML protein accumulation. These studies offer valuable insights into potential cancer treatments based on targeting LLPS and PML NBs.

### 2.3 SGs, CML and myeloma

SGs are cytoplasmic ribonucleoprotein granules that form during cellular stress. SGs are formed via LLPS through a complex network of protein‒RNA interactions with the G3BP1 protein as the central node 49. Various stressors, such as extreme temperature, oxidation, UV radiation, and endoplasmic reticulum or mitochondrial stress, induce the formation of SGs 50. However, the precise mechanisms by which SGs contribute to tumor development and assist cancer cells in coping with tumor-related stress remain incompletely understood 51,52.

CML is associated with chromosomal translocation between the ABL gene on chromosome 9 and the BCR gene on chromosome 22. This genetic abnormality results in the production of the BCR-ABL fusion protein, which leads to persistent activation of the tyrosine kinase activity of BCR-ABL and is the pathophysiological cause of CML. Previous studies have demonstrated the cytoplasmic localization of BCR-ABL; however, its specific subcellular localization remains unclear and disputed 53. Evidence suggests that BCR-ABL proteins are present in granule-like structures in myeloid and lymphoid cells 53,54. These BCR-ABL proteins are predicted to possess long IDRs, which endow weak multivalency and potentially facilitate phase separation and granule formation 55,56. Kashiwagi et al. revealed that BCR-ABL-positive granules were SGs 57. The kinase activity of ABL and the N-terminal region of BCR are crucial for the phase separation of BCR-ABL 57. The granules formed as a result of BCR-ABL fusion play a pivotal role in promoting leukemogenic activity by inhibiting the mRNA translation of the tumor suppressor gene BCRA1 and recruiting downstream signaling proteins 57,58. Consequently, targeting the process of ABL-BCR phase separation could constitute a novel therapeutic strategy for CML.

The receptor tyrosine kinase/RAS/MAP kinase (MAPK) pathway is an oncogenic pathway with widespread aberrancies in human cancers; indeed, 20%-30% of cancer patients harbor RAS (KRAS, HRAS, or NRAS) mutations. Approximately 20% of multiple myeloma patients harbor KRAS mutations, leading to activation of the MEK/ERK pathway. Qiang et al. discovered that mutant KRAS significantly increases SG formation in multiple myeloma cells through 15-deoxy-delta^12^,^14^-prostaglandin J^2^ (15d-PGJ2) 59. Additionally, inhibition of cyclooxygenase-2 (COX2) enhances the sensitivity of KRAS-mutant multiple myeloma cells to bortezomib 59. Therefore, targeting the formation of SGs could constitute an effective approach for treating KRAS-mutant myeloma 59.

### 2.4 N6-methyladenosine (m6A) and myeloid leukemia

m6A is the predominant chemical modification detected on mRNAs and critically influences myeloid leukemogenesis 60,61. However, the precise impact of m6A on different oncogenic processes across cellular contexts remains unknown. YTH domain-containing proteins (YTHDF 1-3), which serve as m6A readers, are involved in mediating the effects of m6A 62. These proteins also possess an N-terminal prion-like low complexity domain (LCD), which is believed to be able to undergo LLPS 63. Ries et al. demonstrated that m6A-binding proteins such as YTHDF1, YTHDF2, and YTHDF3 undergo LLPS facilitated by mRNAs containing multiple m6A-modified bases, both in cells and *in vitro*. During LLPS, mRNAs with multiple m6A modifications act as multivalent scaffolds for m6A-binding proteins and simultaneously bring their LCDs into proximity, facilitating LLPS 64.

Cheng et al. further revealed that the m6A reader protein YTHDC1 undergoes LLPS by binding to m6A, forming nuclear YTHDC-m6A condensates in myeloid leukemia cells. The IDR domain and m6A-binding capability of YTHDC1 are essential for this LLPS, and the abundance of YTHDC-m6A condensates serves as the basis for acute myeloid leukemogenesis (Figure 2C) 65. Given the high prevalence of abnormal m6A modifications in leukemia, targeting m6A and the associated LLPS process may constitute a promising therapeutic approach for myeloid leukemia.

## 3. LLPS in solid cancers

### 3.1 LLPS of fusion proteins in sarcomas

**Figure 3:**
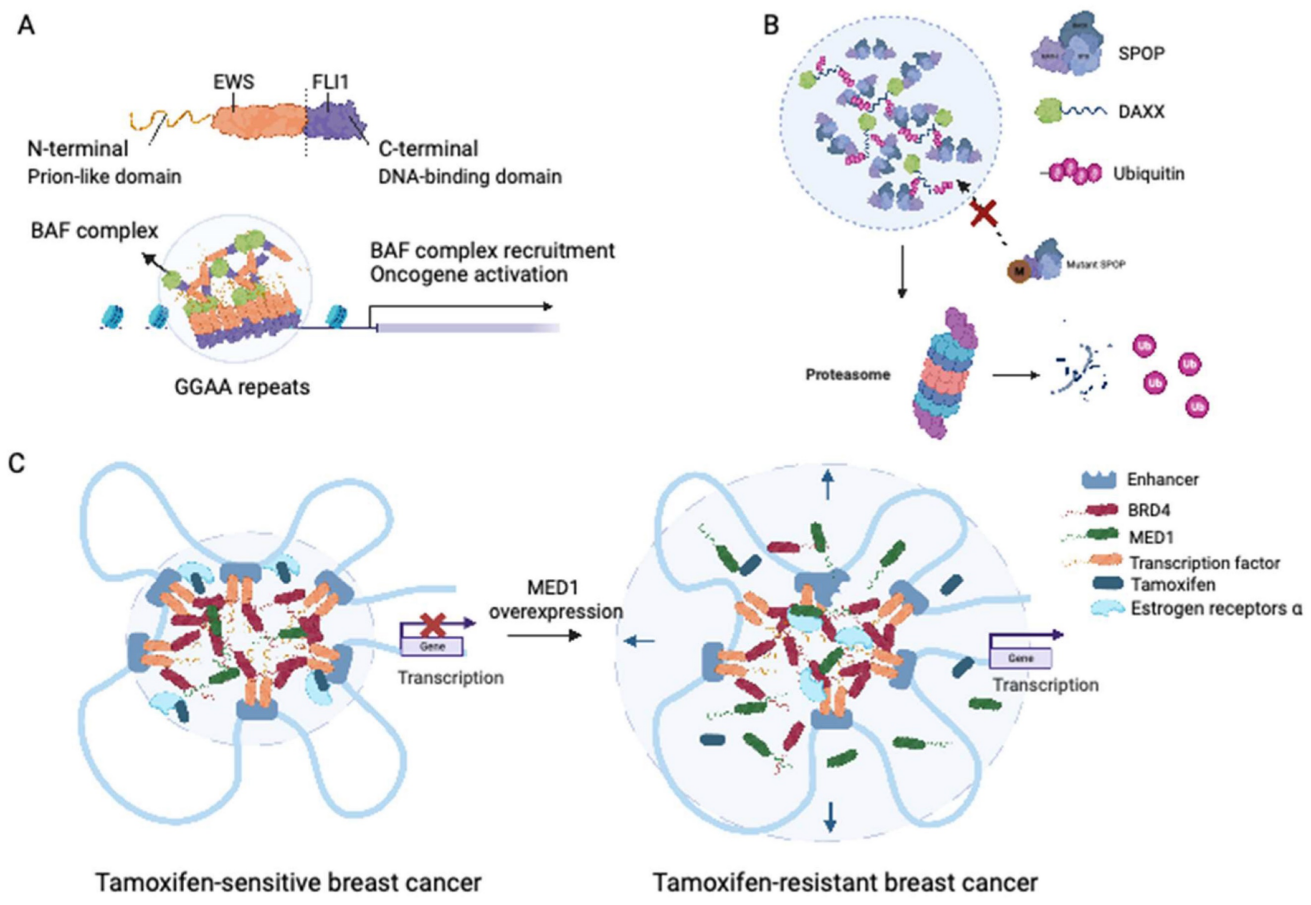
** Roles of LLPS in solid tumor.** (A) EWS-FLI fusions acquire the ability of LLPS in Ewing's sarcoma. EWS-FLI1 fusion protein contains the prion-like domain on the N terminal and the DNA binding domain on the C terminal. The EWS-FLI1 fusion proteins undergo LLPS and form EWS-FLI1 condensates. These condensates then bind to the GGAA microsatellites of DNA, recruiting the BAF complex and acting as super-enhancers to activate oncogenic expression. BAF: BRG1-BRM-associated factor. (B) Mutant SPOP could not form biomolecular condensates in prostate cancer. Wild-type SPOP could self-associate to dimers, interact with the IDR domain of DAXX, and drive the LLPS. The formation of SPOP-DAXX condensates could accelerate the polyubiquitination of DAXX. For mutant SPOP in prostate cancer. For prostate cancers with mutations in the MATH region of SPOP, LLPS cannot proceed, and thus the ubiquitinated degradation of DAXX is terminated. (C) Sensitivity to tamoxifen in breast cancer patients is related to the magnitude of LLPS. In tamoxifen-sensitive breast cancer, MED1, BRD4, together with TFs and RNA polymerase II undergo LLPS and form transcriptional condensates at the site of super-enhancers. With the upregulation of MED1 expression, the volume of the condensate containing MED1 increases and relatively dilutes the tamoxifen concentration in the condensate. MED1: mediator complex subunit 1; BRD4: bromodomain-containing protein 4.

Ewing sarcoma is an aggressive malignancy that predominantly affects children and young adults and primarily involves bones and soft tissue. The presence of the EWS-FLI1 fusion protein, detected in approximately 85% of Ewing sarcoma cases, is considered a distinctive characteristic of this malignancy 66. The fusion protein responsible for Ewing sarcoma is generated by the fusion of the LCD of Ewing sarcoma RNA-binding protein 1 (EWSR1) with the transcription factor Friend leukemia virus integration 1 (FLI1) 67. Notably, fusions of the N-terminal prion-like domains of EWS to FLI1 have been implicated in facilitating protein aggregation through LLPS 68. Boulay et al. demonstrated that the interaction between EWS-FLI1 and BAF chromatin remodeling complexes is dependent on specific tyrosine residues within the prion-like domain of EWSR1 69. LLPS-mediated recruitment of GGAA microsatellites to BAF complexes has been proposed as a mechanism that activates the aberrant transcriptional program driving Ewing sarcoma progression, encompassing processes such as DNA binding, BAF complex recruitment, enhancer activation, and target oncogene activation 69 (see Figure 3A).

In liposarcoma, chromosomal translocations often result in fusion events involving the prion-like domains of other proteins of the FET family and transcription factors 70. It is reasonable to speculate that fusion proteins associated with FUS and TAF15 may promote the formation of abnormal condensates at enhancer or promoter sites, leading to the activation of downstream tumorigenic transcriptional processes. Davis et al. demonstrated that the oncogenic fusion protein FUS-DDIT3 forms condensates through LLPS facilitated by its prion-like domain 71. These nuclear FUS-DDIT3 condensates recruit mammalian SWI/SNF complexes, thereby driving transcription through ectopic chromatin remodeling 71. The same study further indicated that other transcriptional regulators containing prion-like domains can interact with mSWI/SNF chromatin remodelers to orchestrate similar transcriptional programming 71.

Given the notable involvement of FET family members in sarcomas, targeting the aberrant LLPS process mediated by FET fusion proteins could constitute a therapeutic approach. Disrupting the formation of abnormal condensates at enhancer or promoter sites holds promise for inhibiting downstream tumorigenic transcriptional processes facilitated by fusion proteins. This strategy may involve the development of small molecules or therapeutic agents designed to interfere with the LLPS process or disrupt interactions between fusion proteins and their associated complexes. Remarkably, recent studies by Ryan et al. demonstrated that engineered protein disaggregases, such as modified Hsp104 variants, can antagonize the accumulation of FUS-CHOP and EWS-FLI fusion protein condensates formed by LLPS, thus reducing the toxicity of these fusion proteins 68. These findings highlight the immense therapeutic potential of targeting the LLPS process in sarcoma.

Along with FET family members, LLPS has been shown to be involved in the oncogenicity of the SS18-SSX fusion protein. Phase separation of SS18 or SS18-SSX is initiated through self-association of their IDRs, which subsequently facilitates the recruitment of BRG1. Notably, the tyrosine residues within the QPGY domain are crucial for triggering this LLPS process. Disruptions in the LLPS of SS18-SSX or its interaction with BRG1 have been observed to impair the transformation of NIH3T3 cells, highlighting the potential of targeting this LLPS process as a promising therapeutic strategy 72.

### 3.2 Cancer-associated speckle-type pox virus and zinc finger (POZ) protein (SPOP) mutation disrupts protein degradation in prostate cancer

SPOP acts as a substrate adaptor for Cullin 3 RING E3 ubiquitin ligases, functioning as a tumor suppressor by facilitating the degradation of various oncoproteins. These oncoproteins include the androgen receptor (AR), BRD4, death-domain-associated protein (DAXX), PD-L1, and Myc 73. Through its involvement in the ubiquitination process, SPOP regulates various cellular activities, such as cellular metabolism, senescence, and hormone signaling 74. SPOP mutations are observed predominantly in prostate cancer 75. These cancer-associated mutations are frequently located in the MATH domain of SPOP, which is responsible for substrate binding. Consequently, these mutations disrupt the normal interaction with and degradation of substrates, leading to accumulation of the substrates 76,77.

Experimental evidence has demonstrated that SPOP exhibits self-association properties and can form oligomeric structures 78. Bouchard et al. observed the colocalization of SPOP and DAXX in liquid nuclear organelles, and *in vitro*, their binding triggered phase separation mediated by multiple weak SPOP-binding motifs in DAXX 79. Prostate cancer-associated mutations, such as W131F and F133V, within the MATH domain of SPOP have been found to disrupt the interaction between SPOP and DAXX. This disruption interferes with the phase separation process and inhibits substrate ubiquitination (Figure 3B) 79. These findings elucidate that substrate-directed LLPS may be a universal mechanism contributing to proteostasis and that mutations in SPOP can lead to dysregulated ubiquitination activity.

Bouchard et al. also observed that SPOP induces LLPS of AR 79, a critical transcription factor and the main oncogenic driver in prostate cancer. AR activation requires its interaction with the coactivator MED1, which contains IDRs and can undergo LLPS with itself or other transcription factors 80. Zhang et al. demonstrated that ARs are enriched in MED1-dependent condensates, promoting subsequent transcriptional regulation in prostate cancer cell models 81. The DNA-binding domain of full-length AR can bind RNA and drive phase separation in an RNA-dependent manner. However, in AR-v7 variants, the abundance of AR-rich condensates is diminished due to loss of the interaction between the ligand-binding domain and activation function 1 (AF1) of AR, resulting in impaired regulation of AR's transcriptional role in prostate cancer 82. Basu et al. developed a small molecule compound that covalently binds aromatic moieties to cysteines in the activation domain of AR and accumulates with LLPS of AR 83. This compound enhances the degradation of AR and suppresses AR-dependent transcriptional functions, exhibiting significant antitumor activity in prostate cancer models. Recently, through phase separation-based phenotypic screening, Jingjing Xie et al. identified a potential AR inhibitor, ET516, which selectively disrupts AR condensates and inhibits tumor growth in cells harboring resistance mutations in AR 84. These findings highlight the therapeutic potential of designing drugs that target the IDRs in oncogenic transcription factors to regulate their phase separation ability.

### 3.3 KRAS mutation upregulates SG formation in pancreatic cancer

KRAS mutation is a prevalent genetic alteration observed in pancreatic adenocarcinoma, lung adenocarcinoma, and colorectal cancer 85. Approximately 90% of pancreatic cancers harbor KRAS mutations, most commonly the G12D mutation 86,87. The study by Grabocka et al. provided the first evidence that KRAS mutation alone is sufficient to upregulate SG formation. SGs confer advantages on cancer cells by promoting tumorigenesis and progression, offering protection against tumor-related stress 88. The upregulation of SG formation induced by mutant KRAS is mediated by an increase in the level of 15d-PGJ2, a secreted molecule that acts in both cell-autonomous and non-cell-autonomous manners to modulate the expression of its targets 88. The increased abundance of SGs associated with KRAS mutation is linked to stress resistance properties in cancer cells; thus, SGs are potential biomarkers for tumor fitness and drug responses 88.

In pancreatic cancer cells carrying KRAS mutations, the activation of nuclear factor erythroid 2-related factor 2 (NRF2) leads to upregulation of glycolysis, increased glutamine uptake, and reprogrammed glutaminolysis 89. Mukhopadhyay et al. demonstrated that 15d-PGJ2 activates NRF2 and facilitates the formation of SGs. Activation of NRF2 by 15d-PGJ2 upregulates pathways involved in glutamine metabolism, thereby promoting chemoresistance in KRAS-mutant pancreatic cancers 90. Inhibitors of glutaminase, an enzyme involved in glutamine metabolism, can resensitize gemcitabine-resistant pancreatic cancer cells. Therefore, combination treatment with glutaminase inhibitors and chemotherapy holds promise as an effective therapeutic approach for patients with KRAS mutations.

### 3.4 LLPS of MED1 condensates in breast cancer

Estrogen receptor α (ERα)-positive breast cancer is the predominant subtype among hormone receptor-positive breast cancers 91. Treatment with tamoxifen, a selective modulator of ERs, is an effective therapeutic approach for managing ER-positive breast cancer in both pre- and postmenopausal individuals 92. However, the emergence of tamoxifen resistance presents a significant hurdle and is a primary contributor to mortality in breast cancer patients 93.

The IDRs of MED1 and BRD4 play a crucial role in the formation of transcriptional condensates, which concentrate the transcription machinery, at superenhancers (SEs) 94. Following estrogen stimulation, ERα becomes integrated into MED1 condensates 94. Klein et al. demonstrated that in breast cancer cells, ERα associates with MED1 and undergoes condensation in a tamoxifen-sensitive manner 95. Overexpression of MED1 has been linked to tamoxifen resistance and worse survival outcomes in breast cancer patients 96. Tamoxifen treatment drives the dissociation of ERα from MED1 condensates, as the drug selectively enters the condensates and competes with estrogen for binding 96. Upregulation of MED1 increases the volume of MED1-containing condensates, resulting in dilution of tamoxifen within the condensates and reducing the efficiency of ERα dissociation (Figure 3C) 96. These findings suggest that selective partitioning and concentration within condensates formed via LLPS may play a role in the pharmacodynamics of drugs and that alterations in these condensates could contribute to treatment resistance in breast cancer and other cancers.

### 3.5 LLPS of anaplastic lymphoma kinase (ALK) fusions in lung cancer

ALK fusions, particularly the EML4-ALK fusion, are recognized as oncogenic drivers in a subset of non-small cell lung cancers 97. The EML4-ALK fusion protein lacks a transmembrane domain and is localized primarily in the cytoplasm or microtubules 98. This fusion protein, constitutively active as a tyrosine kinase, plays a pivotal role in initiating lung tumorigenesis 99. While extensive studies have explored the oncogenic properties of EML4-ALK, the involvement of LLPS in regulating its function and contributing to lung cancer development has recently gained attention 100,101.

Recent studies by Qin et al. have demonstrated that EML4-ALK variant 1 can form liquid-like condensates in the cytoplasm, with the EML4 region of the fusion protein playing a crucial role in condensate formation 101. Truncation experiments have revealed that the EML4-N fragment alone is sufficient to drive phase separation, while the ALK-C fragment remains dispersed. Moreover, mutations in aromatic residues within the EML4 region significantly impair phase separation, underscoring the importance of these residues in this process. Functional characterization of EML4-ALK condensates has elucidated their role in activating downstream signaling pathways. Disruption of the LLPS process hampers the hyperactivation of key signaling molecules, such as AKT, ERK1/2, and STAT3, which are crucial for promoting cell survival, proliferation, and angiogenesis 101.

In summary, the emerging evidence of LLPS in ALK fusions, particularly EML4-ALK variant 1, emphasizes the significance of this cellular process in the pathogenesis of lung cancer. Understanding the role of LLPS in regulating the function of EML4-ALK and its downstream signaling pathways is expected to contribute to the development of novel therapeutic approaches for lung cancers with ALK fusions.

### 3.6 Glycogen accumulation and LLPS in liver carcinogenesis

Glycogen is the principal storage polysaccharide in hepatocytes, playing a vital role in liver metabolism and being linked to hepatocellular carcinoma development 102. Dysregulation of glycogen metabolism in premalignant liver lesions leads to impaired glycogen degradation and subsequent accumulation 103. The accumulated glycogen undergoes phase separation, leading to the formation of the Laforin-Mst1/2 complex. This complex sequesters the Hippo kinases Mst1/2 within liquid droplets composed of glycogen, thereby removing their inhibitory effect on Yes-associated protein (Yap) 103. Dysregulation of glycogen accumulation and phase separation has significant implications for liver tumorigenesis.

Research by Liu et al. has demonstrated that increased glycogen storage accelerates the development of liver tumors, while reducing the glycogen content decreases tumor incidence 103. Additionally, the interplay among glycogen accumulation, the Hippo signaling pathway, and downstream effectors such as Yap has been associated with liver enlargement and the formation of tumors 103.

These findings highlight the importance of glycogen accumulation and phase separation in liver tumorigenesis. Understanding the mechanisms underlying the dysregulation of glycogen metabolism and its impact on signaling pathways such as the Hippo and Yap pathways yields valuable insights into the development of hepatocellular carcinoma. Targeting these processes could offer new therapeutic strategies for liver cancer.

## 4. LLPS and potentially targetable proteins

### 4.1 LLPS, YAP, and transcriptional coactivator with PDZ-binding motif (TAZ)

LLPS plays an important role in the transcriptional programs regulated by YAP and TAZ, which are crucial regulators of the Hippo pathway 104. Activation of YAP and TAZ is involved in cancer cell survival and metastasis, tumor microenvironment remodeling, and immune evasion 105. However, in certain cases, these proteins have been found to exhibit tumor-suppressive activities in hematological malignancies and colorectal cancer 106-108.

**Figure 4:**
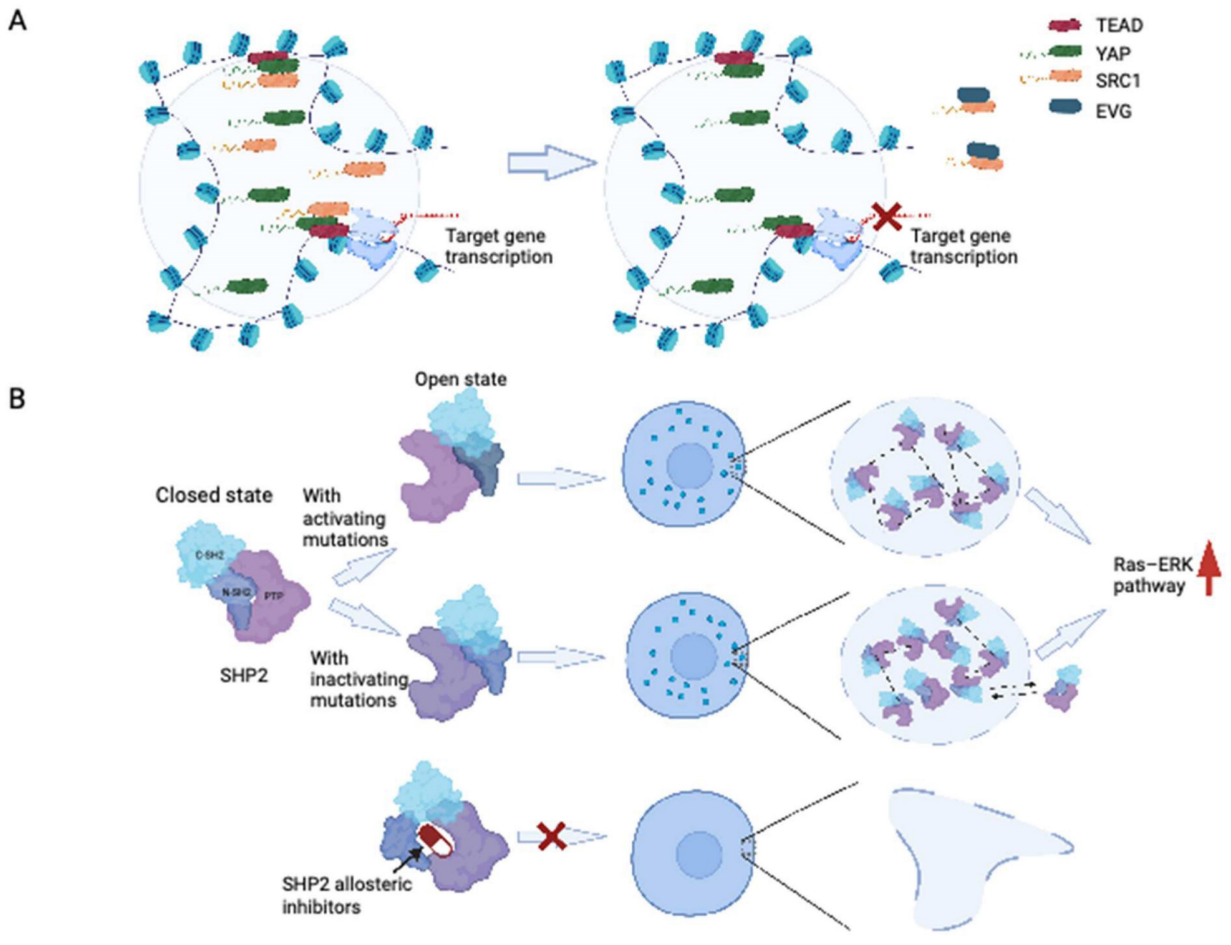
** Disruption of LLPS by targeting related proteins.** (A) Anti-HIV drug elvitegravir could bind to SRC-1 and prevent it from accessing the YAP/TEAD condensates. (B) SHP2 allosteric inhibitors moderate SHP2 mutant undergoing LLPS. Wild-type SHP2 has a well-folded PTP domain which is normally in a closed state. SHP2 with active or inactive mutants could transit the closed states of SHP2 conformation to open states. Mutant SHP2 could boost the activity of PTPase, drive the LLPS, and hyperactivate the Ras-ERK pathway. For SHP2 with active mutation, the high PTPase activity is acquired directly by the upregulated intrinsically PTPase activating, while for SHP2 with inactive mutation, it is mainly acquired from recruiting the wild-type SHP2. SHP2 allosteric inhibitors could stop the formation of SHP2 condensates by keeping SHP2 in closed states. EVG: elvitegravir; PTP: protein tyrosine phosphatase.

Recent research has provided insights into the formation of YAP and TAZ condensates upon exposure to hyperosmotic conditions. The formation of YAP condensates, driven by the IDR transactivation domain, can occur in the nucleus and cytoplasm. These condensates contain TAZ and the transcription factor TEAD1, organized as SEs, and recruit RNA polymerase II to mediate the transcription of YAP target genes 109. Additionally, the interaction between YAP/TEAD and steroid receptor coactivator 1 (SRC-1) leads to the formation of SRC-1/YAP/TEAD condensates, which promote YAP transcription 110. Disruption of SRC-1 condensates by the anti-HIV drug elvitegravir could suppress the proliferation of YAP-dependent tumor cells, showing the potential of targeting phase-separated condensates as a therapeutic strategy 110 (Figure 4A).

In tumor cells, YAP condensates with formation driven by the coiled-coil domain and IDR are associated with anti-PD-1 immunotherapy resistance. These condensates act as transcriptional hubs in the tumor immune microenvironment, concentrating enzymes, transcription factors, and histone acetylases. Disruption of YAP condensates has been correlated with poor treatment outcomes and could be a potential target for combination treatment with immunotherapy 111.

Similar to YAP, TAZ also undergoes LLPS and forms nuclear condensates *in vitro* and *in vivo* 112. TAZ condensates concentrate TEAD4, BRD4, MED1, and CDK9, enhancing the efficiency of transcription and enabling the activation of TAZ-specific downstream pathways. Moreover, unlike YAP, TAZ can form condensates at various protein and salt concentrations and various temperatures, a phenomenon facilitated by its ability to homodimerize through its coiled-coil domain. The Hippo signaling pathway negatively regulates LLPS of TAZ by promoting its phosphorylation via LATS/NDR kinases 112. Furthermore, participation of the paraspeckle protein NONO is required for TAZ phase separation, as it interacts with TEAD and Rpb1. Notably, NONO plays an essential role in TAZ-associated tumorigenesis in glioma, and elevated expression of NONO is correlated with unfavorable survival outcomes in patients with glioblastoma 113.

### 4.2 LLPS and Src homology containing protein tyrosine phosphatase 2 (SHP2)

During cellular processes, protein tyrosine kinases (PTKs) and protein tyrosine phosphatases (PTPs) perform indispensable functions in controlling signaling cascades. SHP2 is a nonreceptor PTP encoded by PTPN11 that dephosphorylates targeted proteins 114. Functionally, SHP2 acts as a central hub connecting various subcellular signaling pathways involved in cancer, such as the RAS/MAPK and PD-1/PD-L1 pathways 115-118. SHP2 dysregulation is associated with several cancers, including breast cancer 115 and melanoma 119. Moreover, gain-of-function mutations in SHP2 are frequently observed in juvenile myelomonocytic leukemia and AML 120.

Recent investigations conducted by Zhu et al. provided important insights into the modulatory role of LLPS in PTP activity, with a specific emphasis on SHP2 121. Researchers have demonstrated that wild-type SHP2 can form biomolecular condensates in response to specific factors such as EGF and FGF, indicating that LLPS may contribute to the regulation of SHP2 activity 120. Notably, cancer-associated SHP2 mutants have a higher propensity for LLPS. LLPS induced by SHP2 mutation represents a gain-of-function mechanism that promotes downstream MAPK signaling, leading to ERK1/2 activation 120. Importantly, Zhu et al. showed that SHP2 mutation-induced LLPS can be suppressed by allosteric inhibitors of SHP2 120 (Figure 4B). These findings are particularly important considering that allosteric inhibitors of SHP2, including AB-3068, TNO155, RMC-4630, and RLY-1971, are currently under evaluation in clinical trials for solid cancers 114. Furthermore, Lin et al. have shown that LLPS could regulate the receptor tyrosine kinase (RTK) signaling through facilitating the formation of a signaling condensate comprising FGFR2, SHP2 and 1-phosphatidylinositol 4,5-bisphosphate phosphodiesterase gamma 1 (PLCγ1) 122. Together, these studies highlight the therapeutic potential of targeting LLPS, particularly by inhibiting SHP2-mediated condensate formation.

### 4.3 LLPS and 53BP1

Genomic instability is a defining characteristic of cancer cells, and preservation of genomic integrity relies heavily on the functionality of DNA damage response pathways 123. A key regulator in this pathway is 53BP1, which acts as a scaffold and facilitates the DNA damage response 124. 53BP1 plays a role in binding to disrupted chromatin, recruiting other DNA double-strand break-responsive proteins, and promoting the synapsis of distal DNA ends during the repair process called nonhomologous end-joining 125. Furthermore, 53BP1 exerts direct regulatory control over the p53 tumor suppressor pathway, which plays a pivotal role in initiating cell cycle arrest, apoptosis, and the activation of other antitumorigenic signaling cascades 125.

Emerging research has substantiated the formation of DNA repair condensates facilitated by 53BP1 via LLPS upon encountering DNA damage, specifically DNA double-strand breaks 126,127. Formation of these condensates is enhanced by long noncoding RNAs that assemble at sites of DNA damage 126,127. The 53BP1 DNA repair condensates serve as platforms for recruiting p53 and its coactivators, leading to stabilization of p53 and facilitating its function 126. Disruption of 53BP1 condensates impairs downstream transcriptional induction and translation of p53 targets, indicating the necessity of these condensates for efficient DNA double-strand break repair 126. Furthermore, Ghodke et al. demonstrated that AHNAK, a component of the USP28-53BP1-p53-p21 network, can modulate G1/S checkpoint functions mediated by 53BP1 and p53 activation on chromatin. Depletion of AHNAK induces the accumulation of 53BP1 and intensifies LLPS, consequently enhancing the activation of the p53 response 128. These findings suggest that disrupting the assembly of 53BP1 condensates could dysregulate p53 and potentially impact the expression of downstream tumor suppressor genes.

Overall, the formation of 53BP1 condensates through LLPS is an important mechanism in DNA repair and p53-mediated cellular responses to DNA damage, and perturbations in these condensates can have significant implications for cancer development. Further exploration into the assembly and modulation of 53BP1 condensates may yield crucial insights for the discovery of novel therapeutic strategies aimed at selectively targeting DNA repair pathways in cancer.

### 4.4 LLPS and PARP family members

PARP1, a nuclear protein, is the first discovered member of the PARP protein family and is involved in poly(ADP-ribose) (PAR) polymerization 129. PARP1 functions as a writer of poly(ADP-ribosyl)ation (PARylation) by catalyzing the addition of ADP-ribose units to form negatively charged PAR chains on itself and other proteins, thereby regulating various pathways 130. PARP1 is indispensable for DNA repair mechanisms, stabilization of DNA replication forks, and alterations in chromatin structure 131. At loci undergoing DNA damage, PARP1 catalyzes the formation of extended PAR chains, thus initiating the assembly of DNA damage condensates through phase separation. This process is facilitated by the specific recruitment of FET proteins containing LCDs 132. Electrostatic interactions between PAR chains and LCDs promote phase separation, while aggregation-prone prion-like domains contribute to the amplification of this response, facilitating DNA repair processes 132.

The advent of PARP inhibitors constituted a substantial breakthrough in anticancer therapeutics, particularly for tumors characterized by impaired homologous recombination repair, including breast, ovarian, pancreatic, and prostate cancers carrying BRCA1/2 mutations 133. However, considering the functional complexity of PARP1, additional drugs that target the phase separation process and DNA damage repair mechanisms mediated by PARP1 could be developed. Further research in this area may provide insights into novel therapeutic strategies for cancer. By understanding and manipulating LLPS of PARP1, it might be possible to increase the effectiveness of PARP inhibitors or develop alternative approaches to target the DNA repair machinery in cancer cells.

## 5. Approaches that target proteins via LLPS

Traditional structure-based drug design relies on well-defined protein structures and pockets, which many oncoproteins, including transcription factors, may not have. Indeed, these proteins often lack folded structures and do not possess clearly defined pockets for drug binding. This limitation poses a substantial challenge in the development of therapies directed against these "undruggable" targets. However, the discovery of LLPS has provided new opportunities for targeting these proteins (Table 2).

### 5.1 Targeting IDRs

Targeting IDRs has emerged as a promising approach for disrupting LLPS and its associated pathogenic condensates. As previously described, IDRs are prevalent in proteins, especially oncoproteins and transcription factors that lack well-defined structures. These IDRs play critical roles in LLPS by mediating interactions between proteins and nucleic acids, contributing to the formation of biomolecular condensates 134.

Small molecule inhibitors that specifically bind to IDRs have shown potential in disrupting LLPS and modulating condensate formation. One approach to target IDRs is the use of small molecule inhibitors. These inhibitors are designed to bind specifically to the IDRs, disrupting their interactions and interfering with the assembly of condensates. For example, to target c-MYC, a well-known oncoprotein, highly specific small molecule inhibitors such as IIA4B20, IIA6B17, and mycmycin-1/2 have been developed. These inhibitors effectively target the IDRs of c-MYC, inhibiting its oncogenic activity and suppressing malignant cell transformation 134,135.

The TFIID transcription complex is a critical component of the transcription initiation machinery in eukaryotes. Within this complex, the TAF2 subunit contains an IDR that plays a regulatory role in transcriptional activation. In an important discovery, a tin-based metal cluster was identified as a selective inhibitor that binds specifically to this IDR within the TAF2 subunit of TFIID. By targeting the IDR, the metal cluster acts as a selective inhibitor, modulating the activity of TFIID and subsequently impacting transcription initiation processes 136.

Recently, a noteworthy compound called PCG, derived from a natural source, has emerged as a potential regulator of LLPS. PCG has been shown to effectively convert phase-separated BRD4 into stable aggregates both *in vivo* and *in vitro* by specifically targeting the IDR of BRD4 137. This targeted action of PCG results in the suppression of BRD4-dependent gene transcription and thus opens a promising avenue for therapeutic intervention.

Another approach to target IDRs is through peptide-based inhibitors. Peptides such as ReACp53 and polyarginine analogs have shown promise in blocking amyloid formation by p53 mutants. These peptides directly interact with the IDRs of p53 mutants, preventing their aggregation and restoring their tumor-suppressive function in cancer cells 138.

### 5.2 Targeting the modification of condensates

The regulation of LLPS dynamics is notably influenced by PTMs, modifications occurring after protein translation. Exploiting these PTMs as intervention targets holds considerable promise. The effectiveness of olaparib, a small molecule inhibitor of PARP1/2, has been demonstrated in terms of its ability to inhibit the formation of condensates involved in PARylation-related DNA repair and disrupt the DNA damage response. By inhibiting PARP activity, these inhibitors modulate the PTMs involved in condensate assembly, thereby impacting their functions and cellular outcomes 139,140. Furthermore, by inhibiting PARP1/2 activity, PARP inhibitors disrupt the PARylation process and alter the dynamics of LLPS. PARylation functions to generate a scaffold to facilitate the recruitment of proteins and nucleic acids to condensates, thereby promoting LLPS. Inhibition of PARP1/2 causes a reduction in the PAR level, affecting the assembly and stability of condensates formed through LLPS 141.

Kinases involved in LLPS regulation are another intervention target. For example, DYRK3, a kinase involved in LLPS, can drive the dissolution of SGs to release mTORC1. By inhibiting DYRK3 activity with GSK-626616, the recondensation process can be promoted in cells. Thus, the disrupted SGs can re-form, leading to sequestration of RNA and proteins within the recondensed granules. Additionally, DYRK3 inhibitors can effectively suppress mTORC1 signaling by preventing the release of mTORC1 from dissolved SGs 142.

Furthermore, the small molecule inhibitor JQ1 has gained considerable attention for its ability to target LLPS. JQ1 acts primarily on BET proteins, particularly BRD4. By binding to the bromodomain of BRD4, JQ1 disrupts the interaction between BRD4 and acetylated lysine residues, thereby interfering with its recruitment to hyperacetylated promoter and enhancer regions 143. Moreover, BRD4 can physically associate with the Mediator complex, and the application of JQ1 can lead to rapid release of Mediator. This dissociation of Mediator from chromatin is strongly correlated with repressed transcription of nearby genes. Notably, these genes exhibit significant enrichment as targets of the MYB transcription factor, a key regulator of leukemogenesis, as well as for functions associated with the development and progression of leukemia 144.

Targeting modifications of condensates is a valuable strategy to modulate their assembly, stability, and function. By specifically interfering with the PTMs involved in LLPS, these approaches can regulate condensate behavior and cellular outcomes. However, it is crucial to consider the specificity and selectivity of these inhibitors to minimize off-target effects and potential disruption of normal cellular processes.

### 5.3 Targeting the drug partitioning process

In traditional approaches, small molecule inhibitors disrupt LLPS by directly interacting with the components of condensates. However, an alternative strategy focuses on the process of drug partitioning into phase-separated condensates, which changes their physicochemical properties and functional outcomes. As Klein et al. reported, several antineoplastic compounds, such as cisplatin, mitoxantrone, tamoxifen, THZ1, and JQ1, can become highly concentrated into biomolecular condensates (e.g., MED1 condensates), which influences their therapeutic activity. This selective partitioning into SGs may occur even in the absence of a compound's targets 95. This group further found that molecules with aromatic rings are more likely to accumulate within MED1 condensates, suggesting that the physicochemical properties of small molecules, such as pi-pi or pi-cation interactions, contribute to their selective partitioning into MED1 condensates 95. In addition, cisplatin treatment was found to induce gradual and specific disruption of MED1 condensates, thus clarifying the mechanisms through which platinum drugs effectively target tumor cells strongly dependent on SE-driven oncogene expression.

### 5.4 Advantages of and challenges in targeting LLPS

Targeting phase separation as a therapeutic strategy offers important advantages in the development of novel interventions (Table 2). By modulating the assembly and dynamics of biomolecular condensates, precise control over cellular processes can be achieved. This approach provides a unique opportunity to restore normal cellular functions and attenuate disease progression. Moreover, targeting phase separation offers a new approach to overcome challenges in traditional drug discovery. Conventional drug development often focuses on inhibition of specific protein targets with well-defined structures. However, many proteins lack well-defined structures and are challenging to target using traditional approaches. By targeting the process of phase separation itself, rather than specific protein structures, a broader range of potential therapeutic targets becomes accessible 145.

However, targeting phase separation also presents challenges. Biomolecular condensates are complex and heterogeneous; thus, understanding their precise mechanisms and identifying specific targets requires comprehensive characterization techniques and advanced computational methods. Achieving selectivity while preserving normal condensates is another challenge, as modulating phase separation may impact essential cellular structures and functions. Robust experimental and computational approaches are crucial for target identification, validation, and optimization of drug candidates.

## 6. Discussion

LLPS has recently emerged as a novel biophysical paradigm, offering valuable insights into the spontaneous formation of membraneless organelles 146,147. The causal relationship between aberrant LLPS and cancer is a crucial issue that needs to be addressed to establish the importance of LLPS in oncogenesis.

As mentioned earlier in this review, evidence has shown that the LLPS-competent IDR in NUP98-HOXA9 plays a pivotal role in leukemogenesis. The IDR facilitates the formation of puncta, which leads to enhanced chromatin occupancy of the chimeric transcription factor and promotes transcriptional activation 27. Other evidence for a causal role of LLPS is that LLPS of SHP2 can be targeted therapeutically using allosteric inhibitors, which attenuate LLPS of SHP2 mutants and enhance the enzymatic activity of SHP2. This observation provides evidence that LLPS can regulate the activity of SHP2, suggesting its potential as a therapeutic target 121. A very recent study conducted by Song et al. further demonstrated that hotspot mutations in the structured Yaf9, ENL, AF9, Taf14, Sas5 (YEATS) domain of the chromatin reader eleven-nineteen-leukemia (ENL) played a pivotal role in the formation of aberrant transcriptional condensates associated with cancer 148. The condensates formed by ENL mutants at physiological levels are functionally associated with upregulation of oncogenes. Moreover, excessive expression of ENL mutants can result in the formation of dysfunctional condensates 148. Collectively, the results of these studies, among others, support the idea that aberrant LLPS plays a causal role in cancer. Through the utilization of mutants with impaired LLPS capacity, investigators have established a direct connection between dysregulated LLPS and the acquisition of oncogenic phenotypes 149.

Understanding LLPS presents an opportunity to design drugs with new targeting strategies. One of the greatest advantages of LLPS is that it presents a novel approach to address the limitations encountered in traditional drug discovery. Conventional drug development revolves primarily around inhibiting specific protein targets with well-defined structures. However, proteins involved in LLPS, such as IDR proteins, often lack distinct structures, posing challenges for conventional targeting methods 145. By directing efforts toward the modulation of phase separation itself, rather than individual protein structures, a wider array of potential therapeutic targets can be explored, circumventing the limitations of traditional approaches. For example, the SRC1 inhibitor elvitegravir disrupts the formation of SRC-1 condensates in cancer cells, presenting a promising LLPS-based strategy for targeting the traditionally challenging SRC-1/YAP/TEAD complex and suppressing YAP-dependent cancer proliferation 110. This approach provides new possibilities for overcoming challenges in drug discovery and expanding the scope of therapeutic interventions. the future development of effective therapeutics requires a comprehensive understanding of the biophysical principles and regulatory mechanisms underlying LLPS.

A key challenge in studying LLPS lies in the development of novel conceptual frameworks, cutting-edge tools, and sophisticated probes that can effectively modulate the physicochemical properties of specific condensates. Methods for studying LLPS have yielded important insights regarding the properties and dynamics of phase-separated condensates. Ensemble experiments, such as imaging and fluorescence techniques, allow the direct measurement of droplet properties over time and the systematic testing of various factors. Single-molecule techniques such as single-molecule Förster resonance energy transfer (smFRET) offer the ability to observe conformational changes and dynamic processes at the molecular level. Nuclear magnetic resonance (NMR) spectroscopy provides atomic-level structural and dynamic information. The development of novel techniques that can provide both high-resolution structural information and real-time dynamic properties of condensates is needed 150. These techniques could involve the integration of complementary methods, such as cryo-electron microscopy 151 and superresolution microscopy 152, to visualize condensate structures at high resolution. Additionally, the combination of single-molecule techniques with NMR or other spectroscopic approaches could impart a more comprehensive understanding of condensate behavior 5. Furthermore, given the intricate complexity and regulatory mechanisms in the intracellular environment, the effectiveness and safety of anticancer agents that modulate LLPS must be evaluated in animal models.

In conclusion, targeting phase separation offers promising opportunities for therapeutic interventions in various diseases, including cancer. This strategy provides a means to disrupt pathological condensates and restore normal cellular function. However, the complexity of LLPS, the dynamic nature of condensates and the need for specificity pose challenges that must be carefully addressed. Future research and innovative approaches are necessary to overcome these challenges and fully exploit the therapeutic opportunities associated with manipulating LLPS for disease treatment.

## Figures and Tables

**Table 1:** Main LLPS-related biomolecules and their roles in different cancer types.

Cancer type	Biomolecule involved in LLPS	Related condensates	Biological role of biomolecules	Reference
**Leukemia and myeloma**				
Myeloid leukemia	NUP98 fusion proteins	Signaling puncta	Promotion of aberrant transcriptional programs	(21,22)
	YTHDC1	SEs	Suppression of myeloid leukemic differentiation	(63,64)
PML	PML-RARα fusion proteins	PML NBs	Suppression of oncogenic pathways	(32,44)
T-cell acute lymphoblastic leukemia	TAL1	SEs	Activation of oncogenic transcriptional programs	(153)
CML	BCR-ABL fusions	SGs	Inhibition of BCRA1 mRNA translation and recruitment of downstream signaling proteins	(57,58)
Myeloma	KRAS	SGs	Increased formation of SGs	(59)
**Solid cancers**				
Ewing sarcoma	EWS-FLI1 fusions	Transcription condensates	Activation of oncogenic transcriptional programs	(67-69)
Liposarcoma	FET family members	Transcription condensates	Activation of oncogenic transcriptional programs	(71)
Synovial sarcoma	SS18	Transcription condensates	Activation of oncogenic transcriptional programs	(72)
Prostate cancer	SPOP and DAXX	Nuclear speckles	Degradation of oncoproteins	(76)
	AR and MED1	SEs	Androgen resistance	(79,81,82)
Pancreatic cancer	KRAS	SGs	Enhancement of tumor cell adaptability through increased SG formation	(88,89)
Breast cancer	MED1 and BRD4	SEs	Activation of oncogenic transcriptional programs; tamoxifen resistance	(94,95)
Lung cancer	EML4-ALK fusion	Signaling puncta	Initiation of lung tumorigenesis	(99,101)
Liver cancer	G6PC	Glycogen compartments	Activation of YAP signaling pathways	(103)
**Multiple cancers**	YAP and TAZ	Transcription condensates	Activation of YAP signaling pathways; anti-PD-1 immunotherapy resistance	(109-111)
	SHP2	Signaling puncta	Stimulation of downstream MAPK and ERK1 signaling pathways	(121)
	53BP1	DNA repair foci	DNA damage repair	(125,128)
	PARP	DNA repair foci	DNA damage repair	(131)
	mTORC1	SGs	Cancer cell survival	(154)
	CDK7, CDK12, CDK13	Transcription condensates	Activation of oncogenic transcriptional programs	(155,156)
	NEAT1	Nuclear paraspeckles	Chemoresistance	(157)

**Table 2:** Strategies targeting LLPS and representative drugs.

Targeting strategy	Representative drugs	Drug targets	Goal of drug targeting	Reference
Modification of condensates	Olaparib	PARP-1; members of the ADP-ribosyltransferase family	Suppression of DNA repair by inhibiting FUS condensate enrichment in regions of DNA damage	(140)
	Chemical inhibitors of BET bromodomains (e.g., JQ1 and IBET)	BET protein BRD4	Inhibition of gene-specific transcriptional activation by releasing the Mediator complex from SEs.	(158)
Alteration of the drug partitioning process	Cisplatin; mitoxantrone; tamoxifen	SEs	Modulation of the characteristics of condensates and influencing drug concentration and efficacy through targeted localization within SE condensates to enhance therapeutic effectiveness	(95)
Targeting of IDRs	YK-4-279, a derivative of the lead compound	Interaction between EWS-FLI1 and RNA helicase A	Inhibiting the proliferation of Ewing sarcoma by induction of apoptosis in tumor cells	(159)
	Tin(IV) oxochloride-derived cluster	IDR within the TAF2 subunit of TFIID	Specifically, disrupts transcription initiation by selectively impairing the function of TFIID	(136)
	PRIMA-1; ReACp53	p53 mutants	Induction of cell cycle arrest in cancer cells with mutant p53 by restoring the native conformation of aggregated mutant p53 proteins	(160,161)
	IIA4B20; IIA6B17	Transcription factor Myc	Neutralization of the oncogenic effects of Myc by disrupting Myc/Max dimerization	(135,162)
	Elvitegravir, an anti-HIV drug	SRC-1, a transcriptional coactivator for nuclear hormone receptors	Suppression of YAP transcriptional activity in cancer cells through inhibition of phase separation of SRC-1 condensates	(163)
	PCG	IDR of BRD4	Suppression of BRD4-dependent gene transcription	(137)
Dissolution of condensate	Sodium arsenate; vinblastine	Microtubules	Disruption of SG formation via inhibition of SG protein transport along microtubules	(164)
	Allosteric inhibitors of SHP2	SHP2 mutants	Suppression of the RAS-MAPK pathway via inhibition of LLPS of SHP2 mutants	(119,121)
	4,4'-Dianilino-1,1'-binaphthyl-5,5'-disulfonic acid (bis-ANS)	LLPS of the TDP-43 LCD	Modulation of dysregulated LLPS: high concentrations inhibit TDP-43 condensate assembly, whereas low concentrations facilitate the formation of liquid droplets.	(165)
	C108	GTPase-activating protein (SH3 domain)-binding protein 2, G3BP2	Interference with breast tumor progression via modulation of SART3 mRNA regulation.	(166)

## Abbreviations

LLPS: liquid‒liquid phase separation
IDRs: intrinsically disordered regions
AML: acute myeloid leukemia
NUP98: NUcleoPorin 98
CML: chronic myeloid leukemia
Lin-HSPC: lineage-negative hematopoietic stem and progenitor cell
PML: Promyelocytic leukemia
NBs: nuclear bodies
APL: acute promyelocytic leukemia
RBCC: RING finger/B-box/coiled-coil
SIM: SUMO-interacting motif
RARα: retinoic acid receptor-α
RA: retinoic acid
SGs: Stress granules
MAPK: MAP kinase
15d-PGJ2: 15-deoxy-delta (12,14)-prostaglandin J (2)
RTK: receptor tyrosine kinase
COX2: Cyclooxygenase-2
m6A: N6-methyladenosine
YTH: YT521-B homology
LCD: low complexity domain
EWSR1: Ewing sarcoma RNA-binding protein 1
ER: Estrogen
FLI1: Friend leukemia virus integration 1
POZ: pox virus and zinc finger
SPOP: Speckle-type pox virus and zinc finger (POZ) protein
DAXX: death-domain-associated protein
AR: androgen receptor
AF1: activator function 1
NRF2: nuclear factor erythroid 2-related factor 2
Erα: estrogen receptor α
YAP: Yes-associated protein
TAZ: transcriptional coactivator with PDZ-binding motif
SRC-1: steroid receptor coactivator 1
SHP2: Src homology containing protein tyrosine phosphatase 2
PTP: protein tyrosine phosphatases
53BP1: p53-binding protein 1
PARP1: Poly(ADP-ribose) polymerase 1
PARylation: poly(ADP-ribosyl)ation
YEATS: Yaf9, ENL, AF9, Taf14, Sas5
ENL: eleven-nineteen-leukemia
